# Han Chinese family with early‐onset Parkinson's disease carries novel compound heterozygous mutations in the *PARK2* gene

**DOI:** 10.1002/brb3.1372

**Published:** 2019-08-06

**Authors:** Ting Huang, Chen‐Yu Gao, Liang Wu, Peng‐Yu Gong, Ji‐Zheng Wang, You‐Yong Tian, Ying‐Dong Zhang

**Affiliations:** ^1^ Department of Neurology, Nanjing First Hospital Nanjing Medical University Nanjing China; ^2^ Department of Endocrine and Metabolic Diseases, The First People's Hospital of Hefei Anhui Medical University Hefei China

**Keywords:** deletion mutations, duplication mutations, early‐onset Parkinson's disease, Parkin gene (*PARK2*)

## Abstract

**Purpose:**

To identify deletions, duplications, and point mutations in 55 previously reported genes associated with Parkinson's disease (PD) and certain genes associated with tremor, spinocerebellar ataxia, and dystonia in a Han Chinese pedigree with early‐onset Parkinson's disease (EOPD).

**Patients and Methods:**

Clinical examinations and genomic analyses were performed on six subjects belonging to three generations of a Han Chinese family. Target region capture and high‐throughput sequencing were used to screen these genes associated with PD, tremor, spinocerebellar ataxia, and dystonia. The multiplex ligation‐dependent probe amplification (MLPA) method was applied to detect rearrangements in *PARK2* exons. Direct Sanger sequencing of samples from all subjects further verified the detected abnormal *PRKRA*, *SPTBN2,* and *ATXN2* gene fragments.

**Results:**

Two family members were diagnosed with PD based on the clinical manifestations, imaging analyses. *PARK2* gene heterozygous deletion of exon 3 and heterozygous duplication of exon 6 were identified in them (II‐3 and 4). A single heterozygous deletion of exon 3 in *PARK2* was detected in II‐5 and III‐10. A single duplication of exon 6 in *PARK2* was detected in I1. Both the heterozygous mutation c.2834G>A (p. R945H) in exon 16 and the heterozygous mutation c.1924 C>T (p. R642W) in exon 14 of the *SPTBN2* gene were identified in II‐3, II‐4, and III‐10. The heterozygous mutation c.2989 C>T (p. R997X) in exon 24 of the *ATXN2* gene was detected in II‐4 and II‐5, and the heterozygous mutation c.170 C>A (p. S57Y) in exon 2 of the *PRKRA* gene was detected in II‐3, II‐4, and III‐10. Other mutations in some genes associated with PD, tremor, spinocerebellar ataxia, and dystonia were not detected.

**Conclusions:**

Novel compound heterozygous mutations were identified in a Han Chinese pedigree and might represent a cause of EOPD.

## 1 INTRODUCTION

Parkinson's disease (PD) is the second most common progressive degenerative disorder of the central nervous system after Alzheimer's disease (Corti, Lesage, & Brice, 2011; Mason, Ziemann, & Finkbeiner, 2014). PD is clinically characterized by motor symptoms, such as bradykinesia, resting tremor, rigidity, and postural instability, as well as nonmotor symptoms, such as hyposmia, sleep disorders, autonomic nervous system, and psychiatric symptoms. The pathological hallmarks of PD are the loss of midbrain dopaminergic neurons in the substantia nigra, resulting in a significant decrease in the number of dopaminergic neurons in the striatum (Hornykiewicz, 2008), and the presence of Lewy bodies in the cytoplasm of neurons (Pollanen, Dickson, & Bergeron, 1993). Risk factors, such as genetic factors, environmental factors, aging, and oxidative stress, all play important roles in dopaminergic neurons loss, but the mechanisms of these factors' interactions have not been thoroughly elucidated (Dexter & Jenner, 2013; Jiang, Sun, & Chen, 2016).

The incidence of PD increases with age, affecting greater than 1% of the population over the age of 60 and greater than 4% of the population over the age of 80 (Nussbaum & Ellis, 2003). Although most patients with PD exhibit the sporadic form, 5%–10% of patients inherit the disease in a Mendelian pattern (Lesage & Brice, 2009; Lill, 2016). *PARK2* is the most common genetic cause of early‐onset Parkinson's disease (EOPD). Fifty percent of familial autosomal recessive early‐onset parkinsonism cases (≤40) are derived from homozygous or compound heterozygous mutations in *PARK2* (Lucking et al., 2000). *PARK2* is located on chromosome 6q25.2‐27 and contains 12 exons separated by large intronic regions, encoding an E3 ubiquitin ligase named Parkin that consists of 465 amino acids (Asakawa et al., 2001; Matsumine et al., 1997; Shimura et al., 2000). The ubiquitin proteasome system (UPS) is an essential posttranslational modification involved in nearly every cellular pathway. Parkin's primary role in the UPS includes attachment of ubiquitin to a target protein to facilitate its subcellular localization or tag it for degradation (von Coelln, Dawson, & Dawson, 2004; Fiesel et al., 2015; Panicker, Dawson, & Dawson, 2017). The characteristics of EOPD caused by mutations in *PARK2* include typical clinical symptoms of PD, such as bradykinesia, rigidity, and resting tremor, and other features, such as an early onset at an age <40 years, a slower disease progression, a marked and sustained response to levodopa, levodopa‐induced motor fluctuations, dyskinesias, foot dystonia, hyperreflexia of the lower extremities, symptoms of diurnal fluctuations with sleep benefits, and psychiatric symptoms (Grunewald, Kasten, Ziegler, & Klein, 2013; Khan et al., 2003; Schrag & Schott, 2006).

In this study, we reported a Han Chinese family carrying novel compound heterozygous mutations (heterozygous deletion of exon 3 and heterozygous duplication of exon 6) in the *PARK2* gene, as revealed by next‐generation sequencing. In addition, we collected the clinical data from two family members with EOPD and analyzed the family pedigree by assessing samples from six members from three generations.

## 2 MATERIALS AND METHODS

### 2.1 Subjects

Six subjects from a third‐generation Han Chinese family from Anhui Province were investigated in our study. Based on the subjects’ clinical manifestations and physical examinations, two family members were diagnosed with EOPD in Nanjing First Hospital, Nanjing Medical University; other family members did not exhibit clinical manifestations of PD. According to the UK Parkinson's Disease Society Brain Bank criteria, these patients with EOPD were evaluated by two experienced neurologists. All participants provided informed consent, and the study was approved by the ethics committee of Nanjing First Hospital. Our study was conducted strictly in accordance with the implementation rules of the regulations on the administration of medical institutions issued by the State Council of the People's Republic of China.

### 2.2 Genomic and molecular analyses

A whole‐genome library was established as follows: We collected 5 ml of peripheral blood from each of the six subjects into EDTA‐anticoagulant tubes and used a QIAamp DNA Mini Kit (Qiagen) to isolate DNA from these samples. Next, 55 genes previously reported to be associated with PD and other genes associated with tremor, spinocerebellar ataxia, and dystonia were selected for a gene‐capture strategy using the Gen Cap custom enrichment kit (My Genostics Inc.) according to the manufacturer's protocol. PCR was then used to amplify the captured DNA fragments.

For each subject, high‐throughput sequencing of the 55 previously reported PD‐related genes and some genes associated with tremor, spinocerebellar ataxia, and dystonia was conducted using an Illumina HiSeq X10 sequencer (Illumina). All mutations identified by HiSeq X10 sequencing were confirmed by performing Sanger sequencing, including point mutations in the *PRKRA* gene, *SPTBN2* gene, and *ATXN2* gene. The multiplex ligation‐dependent probe amplification (MLPA) method was employed to detect rearrangements in *PARK2* exons and analyze deletions and duplications. DNA sequencing data were analyzed using the Burrows‐Wheeler Aligner (http://bio-bwa.sourceforge.net/) and Genome Analysis Toolkit software 3.7 (https://software.broadinstitute.org/gatk/).

### 2.3 Brain imaging

The proband (II‐3) and her older brother (II‐4) were subjected to 3.0T magnetic resonance imaging (MRI) (Ingenia, Philips Medical Systems) and positron emission computed tomography imaging (PET/CT) (uMI 780, Shanghai United Imaging Healthcare Co., Ltd.) using a 3D acquisition mode. When studied in a PET/CT System, they fasted for 6 hr and were received ^18^F‐DOPA (4 MBq/kg, Jiangsu Huayi Chemical Co., Ltd.) intravenously. To exclude interference between the two imaging agents, ^18^F‐FDG (4 MBq/kg, Andike Pharmaceutical Group Co., Ltd., Ltd.) was administered intravenously the next day under the same conditions. After 90 min from administration of ^18^F‐DOPA and ^18^F‐FDG, a 30‐min static brain scan was performed according to the standard operating procedures protocol of the Nanjing First Hospital, and images were reconstructed with CT‐based attenuation correction.

## 3 RESULTS

### 3.1 Family pedigree map

**Figure 1:**
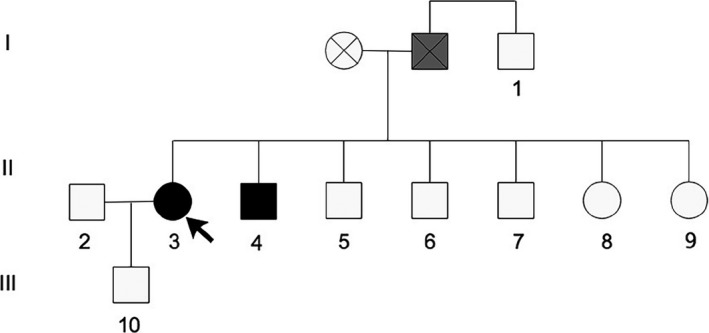
Pedigree map of Parkinson's disease (PD) family. Circle: female; square: male; black: patient; gray: possible patient; circle with a fork: dead female; square with a fork: dead male. The black circle indicated by the arrow represents the proband

An Anhui family with a history of EOPD presented novel compound heterozygous mutations in the *PARK2* gene and carried other mutations in the *PRKRA*, *SPTBN2,* and *ATXN2* genes. The family pedigree was established based on gene sequencing and clinical information for three generations. Two members showed clinical manifestations of EOPD (Figure 1II‐3, 4); the others were unaffected at the time of the study (Figure 1I‐1, II‐2, 5, 6, 7, 8, 9, and III‐10).

### 3.2 Clinical features

In 2002, when she was 37, the 54‐year‐old proband (II‐3) initially manifested signs of resting tremor and an ache in the right limb; she gradually developed rigidity and bradykinesia. Cold or heat stimulus, nervousness, and excitement contributed to the exacerbation of these symptoms. Three years later, these symptoms gradually worsened. She was then diagnosed with PD by neurologists according to the UK Parkinson's Disease Society Brain Bank criteria and administered a small dosage of levodopa and benserazide hydrochloride tablets (125 mg bid); the dosage gradually increased to 125 mg tid combined with trihexyphenidyl (1 mg bid) because the resting tremor extended to all four limbs. However, her 52‐year‐old brother (II‐4) initially manifested signs of resting tremor of the left limb in 1988, when he was 21 years old. He was diagnosed with PD in 2005 when he developed bradykinesia and was administered the same dosage of levodopa and benserazide hydrochloride tablets as his sister. With the slow progression of PD symptoms, he gradually developed postural instability and rigidity. Currently, the patient is being treated with levodopa and benserazide hydrochloride tablets (125 mg bid) and trihexyphenidyl (2 mg bid). Other members were unaffected at the time of the study. The proband and her brother (II‐4) are both sensitive to levodopa. In addition, their blood copper and ceruloplasmin levels were normal, and K–F rings were not observed. The main clinical symptoms of the two patients are summarized in Table 1.

**Table 1:** Clinical symptoms of two patients

Patient ID	II‐3	II‐4
Age at disease onset (years)	37	21
Disease duration (years)	17	31
Sensitivity to dopamine	+	+
Bradykinesia	+	+
Resting tremor	+	+
Rigidity	+	+
Postural instability	−	+
Asymmetry at onset	+	+
Autonomic dysfunction	−	−
Rapid eye movement sleep behavior disorder	−	−
Olfactory disorder	−	−
Numbness	+	−
Hallucination	−	−
Wearing‐off	+	−
Dystonia	+	+
UPDRS score	28	34
Hoehn & Yahr	2.5	3
MOCA score	19	27
MMSE score	22	25
HAMD score	8	2

Abbreviations: HAMD, Hamilton depression scale; MMSE, Mini‐mental State Examination; MOCA, Montreal Cognitive Assessment; UPDRS, The unified Parkinson's disease rating scale.

### 3.3 Brain imaging

**Figure 2:**
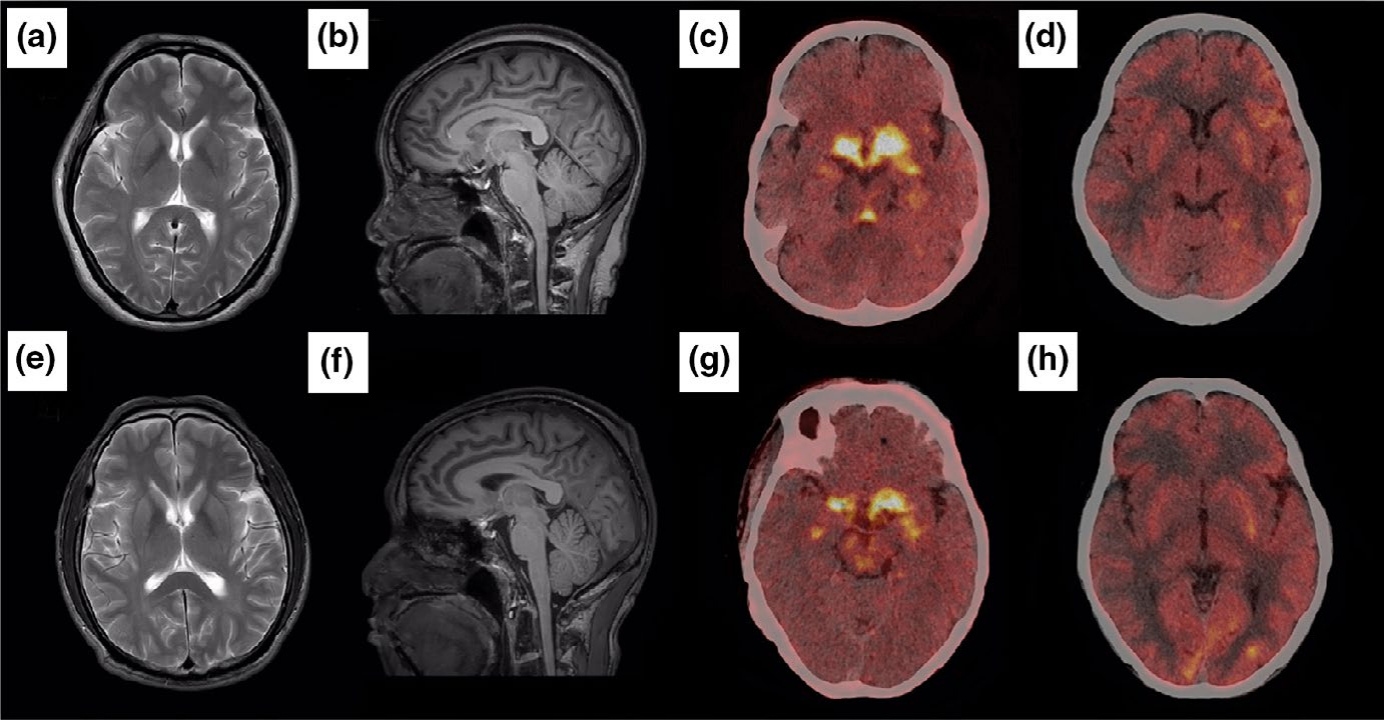
Images of the brains of II‐3 (a‐d) and II‐4 (e‐h) obtained using 3.0T magnetic resonance imaging (MRI), ^18^F‐DOPA, and ^18^F‐FDG positron emission computed tomography imaging (PET/CT)

The brain MRI, ^18^F‐FDG imaging, and ^18^F‐DOPA imaging of two patients revealed a PD‐related pattern. The MRI of II‐3 (Figure 1a,b) showed a few ischemic lesions in the bilateral frontal lobe and parietal cortex, with no other abnormalities. Her ^18^F‐DOPA imaging revealed reduced ^18^F‐DOPA uptake, particularly in the putamen and caudate nucleus, more so on the right than the left (Figure 1c). In addition, ^18^F‐FDG imaging showed an unequal distribution of radioactivity. Decreased ^18^F‐FDG metabolism was observed in the thalamus, putamen, cerebellum, and parietal, temporal, and occipital lobes (Figure 2d). Moreover, the decrease in ^18^F‐FDG metabolism observed in the right hemisphere was significantly greater than that observed in the left hemisphere. Similar distributions of radioactive tracers were detected in one of the brothers of the proband (II‐4) (Figure 2e‐h).

### 3.4 Mutation analysis

**Figure 3:**
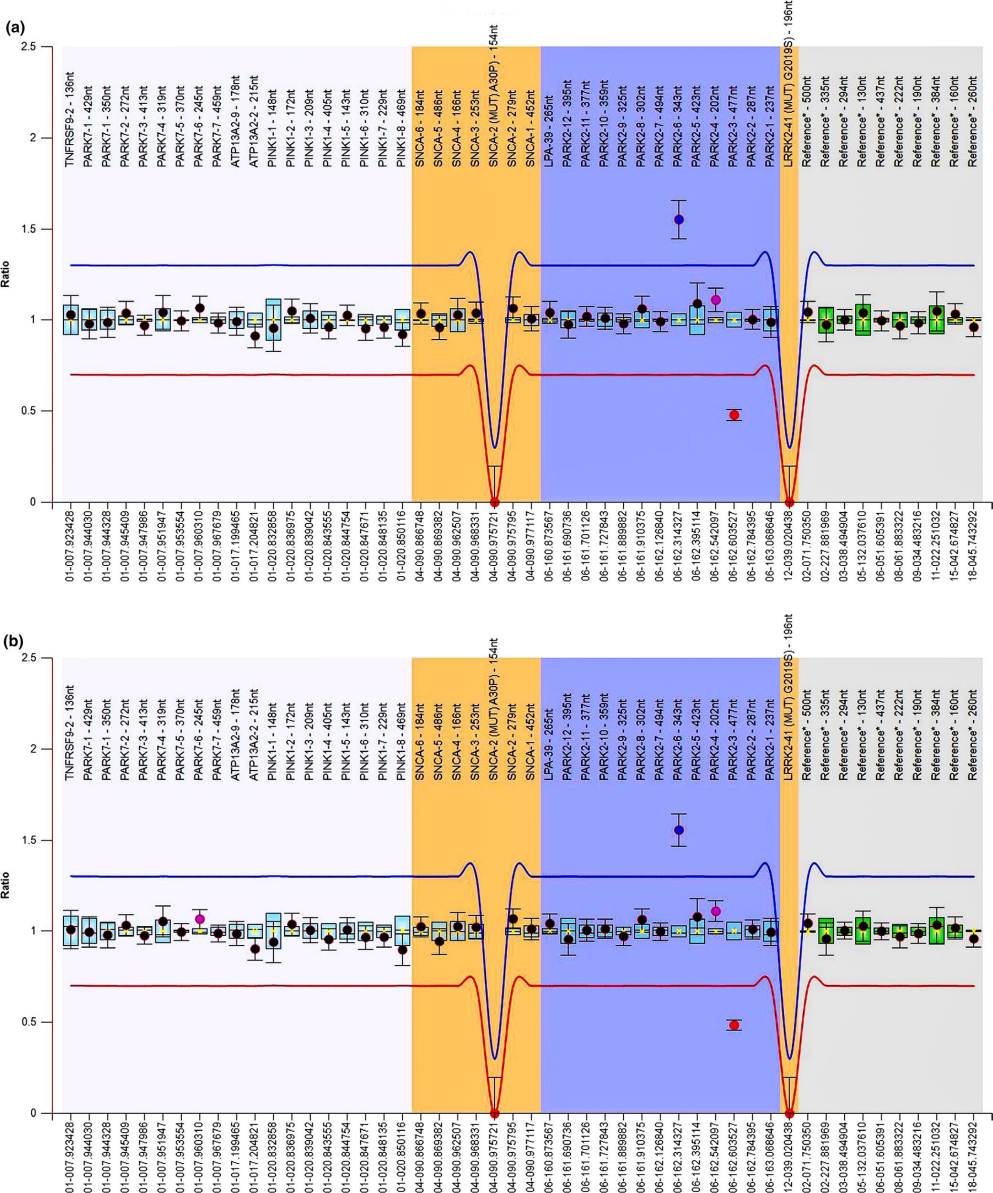
(a) Box chart of II3. (b) Box chart of II4. 0.75–1.25 was considered a normal range; 0.5: heterozygous deletion mutation; 0: homozygous deletion mutation. The blue dot indicates a fluorescence signal intensity of 1.5; the red dot indicates a fluorescence signal intensity of 0.5

Heterozygous deletion of exon 3 and heterozygous duplication of exon 6 in the *PARK2* gene were detected using the multiplex ligation‐dependent probe amplification (MLPA) method. The results for *PARK2* gene sequencing are presented in a box chart, showing that a fluorescence signal intensity <0.75 represents a deletion (0.5: heterozygous deletion mutation, 0: homozygous deletion mutation) and a fluorescence signal intensity >1.25 represents a duplication (0.75–1.25 was considered a normal range). A heterozygous deletion of exon 3 was identified in the proband (II‐3), her two brothers (II‐4 and II‐5), and son (III‐10), for whom a fluorescence signal intensity of 0.5 was observed. In addition, the heterozygous duplication of exon 6 was detected in the proband (II‐3), her older brother (II‐4), and her uncle (I‐1), for whom a fluorescence signal intensity of 1.5 was observed. (Figure 3).

Mutations in the *SPTBN2*, *ATXN2*, and *PRKRA* genes were detected using Sanger sequencing. The proband (II‐3), her older brother (II‐4), and son (III‐10) carried both the heterozygous mutation c.2834G>A (p. R945H) in exon 16 and the heterozygous mutation c.1924 C>T (p. R642W) in exon 14 of the *SPTBN2* gene. In addition, the heterozygous mutation c.2989 C>T (p. R997X) in exon 24 of the *ATXN2* gene was detected in two younger brothers of the proband (II‐4 and II‐5), and the heterozygous mutation c.170 C>A (p. S57Y) in exon 2 of the *PRKRA* gene was detected in the proband (II‐3), her older brother (II‐4), and son (III‐10) (Table 2).

**Table 2:** Mutation analysis of the family pedigree

	*PARK2*		*SPTBN2*		*ATXN2*	*PRKRA*
	Heterozygous deletion mutation of exon 3	Heterozygous duplication of exon 6	Heterozygous mutation c.2834G>A of exon 16	Heterozygous mutation c.1924C>T of exon 14	Heterozygous mutation c.2989C>T of exon 24	Heterozygous mutation c.170C>A of exon 2
I‐1	−	+	−	−	−	−
II‐2	−	−	−	−	−	−
II‐3	+	+	+	+	−	+
II‐4	+	+	+	+	+	+
II‐5	+	−	−	−	+	−
III‐10	+	−	+	+	−	+

## 4 DISCUSSION

As shown in the present study, compound heterozygous mutations in the *PARK2* gene (heterozygous deletion of exon 3 and heterozygous duplication of exon 6 in the *PARK2* gene) caused PD in this Han Chinese family. In addition, the family carried other mutations in the *SPTBN2*, *ATXN2,* and *PRKRA* genes, though family members carrying mutations in these genes did not display corresponding clinical symptoms.

In 1998, Kitada et al. identified deletion mutations in the *PARK2* gene that were responsible for autosomal recessive juvenile parkinsonism (AR‐JP) (Kitada et al., 1998). Subsequently, a diverse range of mutations in this gene have been described, including missense mutations, nonsense mutations, truncations, deletions, duplications, and frameshifts (Hedrich et al., 2004; Mata, Lockhart, & Farrer, 2004). These mutations can be present in homozygous, heterozygous, and compound heterozygous states. Exon rearrangements, including deletions and duplications, account for more than 50% of the pathogenic variations and are more likely to be pathogenic variants than are point mutations or small insertions/deletions, leading to an earlier onset of symptoms (Pankratz et al., 2009).

Heterozygous and homozygous deletions in exon 3 of the gene are common in patients with PD in the Han Chinese population. Guo et al. used fluorescence semiquantitative PCR to analyze exon rearrangements in this gene in 61 sporadic patients with early‐onset parkinsonism and identified a heterozygous deletion of *PARK2* gene exon 3 in one patient with PD (Guo et al., 2006). Furthermore, Guo et al. studied the prevalence of mutations in four genes (*PARK2*, *PINK1*, *DJ‐1*, and *ATP13A2*) in 29 unrelated Chinese families with AREP and found that 10 patients with PD carried a heterozygous deletion of exon 3 in this gene (Guo et al., 2008). Wang et al. also reported a heterozygous deletion of exon 3 in this gene in probands and their relatives in three Chinese pedigrees of AREP (Wang, Ma, Feng, Xie, & Chan, 2010). Although Lee et al. screened 68 probands in Taiwan with early‐onset parkinsonism for mutations in the *PARKIN*, *DJ‐1*, and *PINK1* genes and identified nine patients who harbored mutations in *PARK2,* including heterozygous duplication of exon 6 (Lee et al., 2009), the present study is the first to identify a heterozygous duplication of exon 6 in the population of the Chinese mainland. In Japan, a patient with a positive family history was reported to carry compound heterozygous deletion (deletion of exons 3–5) and duplication (duplication of exons 3–7) mutations (Funayama et al., 2012). In addition, the compound heterozygous deletion (deletion of exons 3–4) and duplication (duplication of exons 6) mutations were detected in one patient with EOPD in South Korea (Kim et al., 2012). The novel compound heterozygous mutations in *PARK2* reported in the present study include the heterozygous deletion of *PARK2* exon 3 and heterozygous duplication of exon 6, without any other known PD‐related mutations in the Han Chinese family. Although Asian populations have similar genetic mutations, the degree of exon rearrangements in the above Japanese and Korean pedigrees exceeded those in the Chinese pedigree we reported. Based on these findings, racial heterogeneity and regional differences exist among the *PARK2* mutations.

Heterozygous mutations of the brain spectrin gene *SPTBN2*, which encodes the protein β‐III‐spectrin, are one cause of spinocerebellar ataxia type 5 (SCA5) (Ikeda et al., 2006), an autosomal dominant neurodegenerative disease. Trinucleotide CAG expansions of the ataxin‐2 protein, encoded by the *ATXN2* gene, increase the susceptibility to amyotrophic lateral sclerosis; in contrast, prolonged expansion leads to spinocerebellar ataxia‐2 (SCA2), an autosomal dominant neurodegenerative disease (Chio et al., 2015; Pulst et al., 1996). The *PRKRA* gene encodes an interferon‐inducible double‐stranded RNA‐dependent activator that when mutated causes DTY16, an autosomal recessive combined dystonia syndrome (Bragg, Armata, Nery, Breakefield, & Sharma, 2011). However, the heterozygous mutations in the *PRKRA*, *SPTBN2,* and *ATXN2* genes confirmed using Sanger sequencing have not yet been reported (Table 2).

We analyzed family members in the current study and found that two patients also carried compound heterozygous mutations (*PARK2* gene heterozygous deletion of exon 3 and heterozygous duplication of exon 6) and that three unaffected members carried single heterozygous mutations. The compound heterozygous mutation in the *PARK2* gene was first identified in the proband (II‐3), who was diagnosed with EOPD at age 37. A carrier of the same compound heterozygous mutation carrier (II‐4) also presented classical PD symptoms, such as resting tremor, postural instability, and myotonia, with an onset age of 21 years. The onset age of the two patients was <40 years old, and both met the age criterion for EOPD. The difference in ages was large, though studies have shown that age at onset can vary greatly, even among individuals with the same pathogenic variant (Chien et al., 2006). Despite carrying the same single heterozygous deletion of exon 3, the younger brother (II‐5) and son (III‐10) of the proband showed no clinical manifestations of PD. Similarly, the uncle of the proband (I‐1) who carried a single heterozygous duplication of exon 6 had no PD symptoms. Currently, the roles of heterozygous mutations in disease development remain a matter of debate and are worthy of study. Although carriers of heterozygous mutations have been reported to suffer from parkinsonism because of reduced expression and enzymatic activity of parkin (Klein, Lohmann‐Hedrich, Rogaeva, Schlossmacher, & Lang, 2007), *PARK2* appears to exert a dose‐dependent effect on the risk of parkinsonism. Therefore, a single heterozygous mutation may not directly lead to PD, as some members of this EOPD family who carried a single heterozygous mutation were not affected (I‐1, II‐5, and III‐10); in contrast, double‐mutation carriers develop PD at a significantly earlier age than do single‐mutation carriers or patients without mutations (Kay et al., 2010). The proband's deceased father experienced a mild right‐hand resting tremor at the age of 50, but his symptoms had not progressed significantly for 20 years. The father did not receive a clear diagnosis of PD. Based on genetic analyses of the family pedigree, we speculate that the father and mother of the proband were likely to be carriers of different single heterozygous mutations. Additionally, heterozygous mutations are likely to cause late‐onset PD. Furthermore, the clinical phenotypes of SCA5, SCA2, and DTY16, which may also be present in some patients with PD, are not currently observed in carriers. Mutations at related sites were analyzed in strict accordance with the ACMG guidelines, and their pathogenicity is uncertain.

In addition, the ^18^F‐DOPA imaging of parkin patients showed a significantly slower loss of ^18^F‐DOPA uptake in the putamen compared with idiopathic Parkinson's disease (IPD) patients whose disease severity was similar to parkin patients. These results suggest that disease progression in patients with parkin mutations is more slowly than IPD patients. Similarly, reduction in ^18^F‐DOPA uptake in bilateral putamen was more obviously than the caudate nucleus. Moreover, brain imaging was performed in only two PD patients in this case. Brain imaging is helpful in the diagnosis and differential diagnosis of PD. Brain imaging of unaffected family members may predict their possibility of developing PD, but the absence of brain imaging does not affect the results of mutation analysis.

To our knowledge, this study is the first to report a compound heterozygous mutation of the *PARK2* gene in a large Chinese family with EOPD. However, there were some limitations in this study. We were unable to collect blood samples from the proband's father and mother, and four family members refuse to participate. We will continue to monitor all members of this Han Chinese family.

In summary, we present novel compound heterozygous mutations in the *PARK2* gene, consisting of a heterozygous deletion of exon 3 and a heterozygous duplication of exon 6 in the present study. The results suggest that these mutations are responsible for EOPD in the Han Chinese population. We hope that this pedigree will provide further insight into EOPD caused by compound heterozygous mutations in the *PARK2* gene.

## CONFLICT OF INTEREST

The authors report no conflicts of interest in this work.

## Data Availability

The data that support the findings of this study are available from the corresponding author upon reasonable request.
